# Supplementary material for: The Significance of the Location of Mutations for the Native-State Dynamics of Human Lysozyme
Source: Biophys J. 2016 Dec 6;111(11):2358–67. doi: 10.1016/j.bpj.2016.10.028 (PMC5153563; doi:10.1016/j.bpj.2016.10.028)
Supplement: Document S1. Figs. S1–S4 and Table S1 [file mmc1.pdf]

Biophysical Journal, Volume *111*

Supplemental Information

The Significance of the Location of Mutations for the Native-State Dynamics of Human Lysozyme

Minkoo Ahn, Christine L. Hagan, Ana Bernardo-Gancedo, Erwin De Genst, Francisco N. Newby, John Christodoulou, Anne Dhulesia, Mireille Dumoulin, Carol V. Robinson, Christopher M. Dobson, and Janet R. Kumita

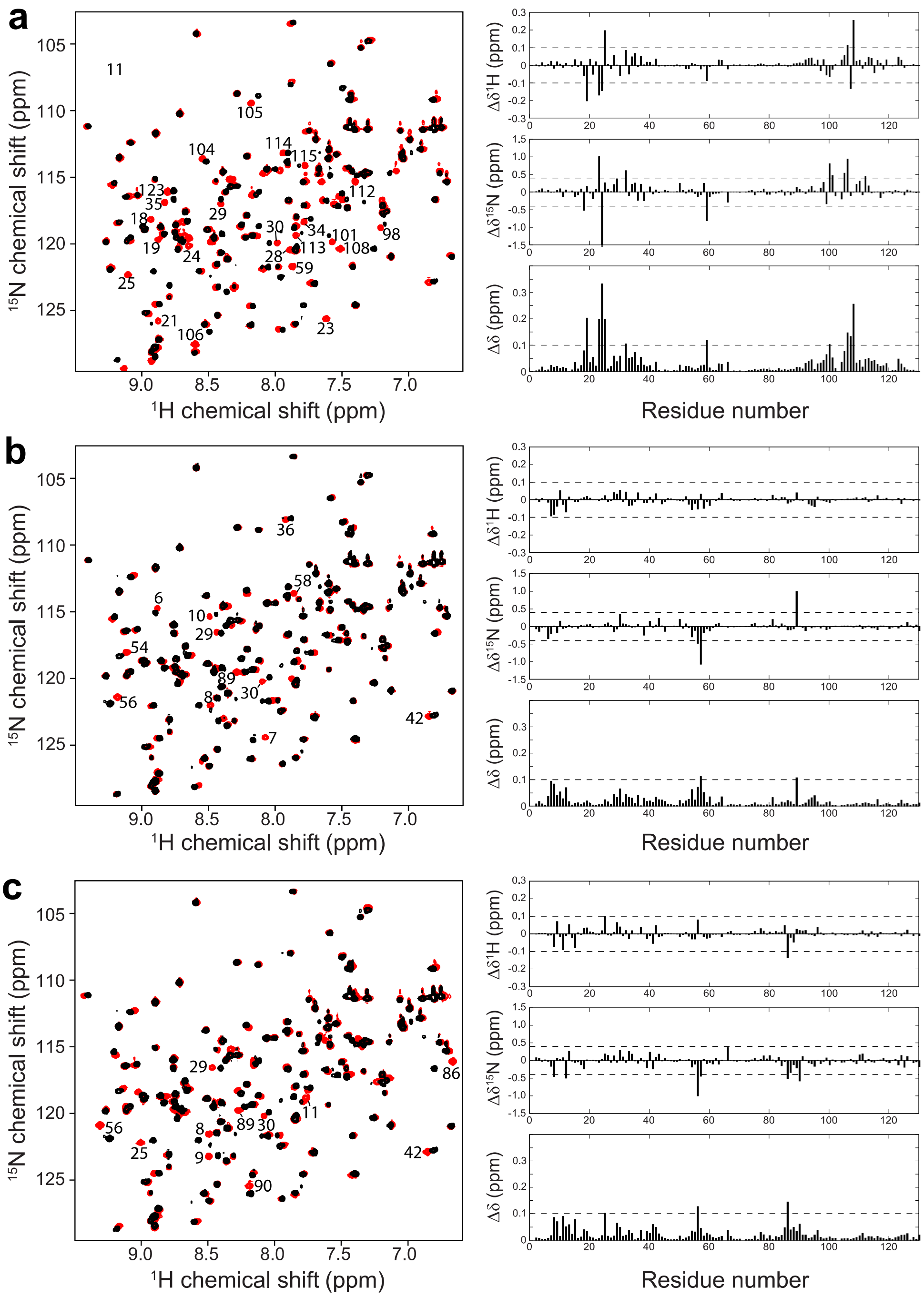

Figure S1. (a), (b), (c) HSQC NMR spectra of I23A (a), I56V (b) and I89V (c) variants (red) overlaid with that of WT lysozyme (black). The spectra were collected at 700 MHz at pH 5.0 and 37 °C. Differences between chemical shift perturbation of each variant and that of WT are shown on the right. $\Delta\delta^{15}N$ (top), $\Delta\delta^{1}H$ (middle) and weighted difference ($\Delta\delta$, bottom) are shown against residue number. Threshold values of ±0.3, ±1.5, ±0.1 for $\Delta\delta^{15}N$, $\Delta\delta^{1}H$ and $\Delta\delta$ are shown in dashed lines. Relative gyromagnetic ratio of ^{15}N and ^{1}H is used as the weighting factor ($\omega = \gamma_{15N}/ \gamma_{1H}$) for calculating $\Delta\delta = ((\Delta\delta^{1}H)^2 + (\omega\Delta\delta^{15}N)^2)^{0.5}$.

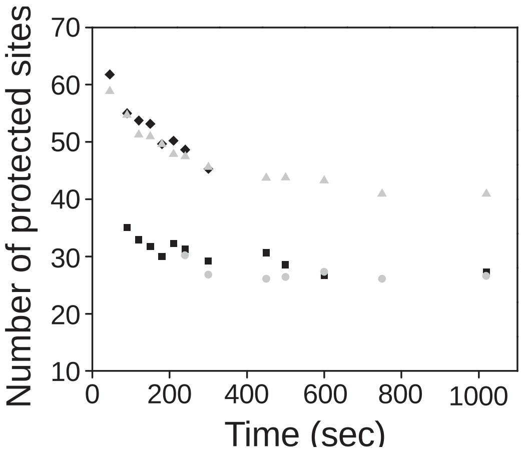

Figure S2. Comparison of the number of protected sites in the higher and lower mass species observed by EX1 HD exchange mass spectrometry for I23A (light grey circles and triangles) and I59T (black diamonds and squares) at pH 8.0, 37°C. Lysozyme variants were deuterated all exchangeable sites and the fully deuterated I23A and I59T had molecular masses of 14912.6 Da and 14943.6 Da respectively. At each time point, the number of exchangeable sites for each mass species was determined (deuterated molecular mass – observed molecular mass). The number of protected sites, i.e. not accessible to the solvent for deuterium-to-hydrogen exchange, for each mass species can be determined (calculated number of exchange sites in variant – observed number of exchanged sites). The calculated number of exchange sites for I23A and I59T are 262 and 263, respectively. Although structural details of the two species cannot be determined under these experimental conditions, it is clear there is close agreement in the number of protected sites for the I23A and I59T variants for both the higher and lower mass species, indicating similar degrees of protection from solvent exchange under EX1 conditions (37°C, pH 8.0).

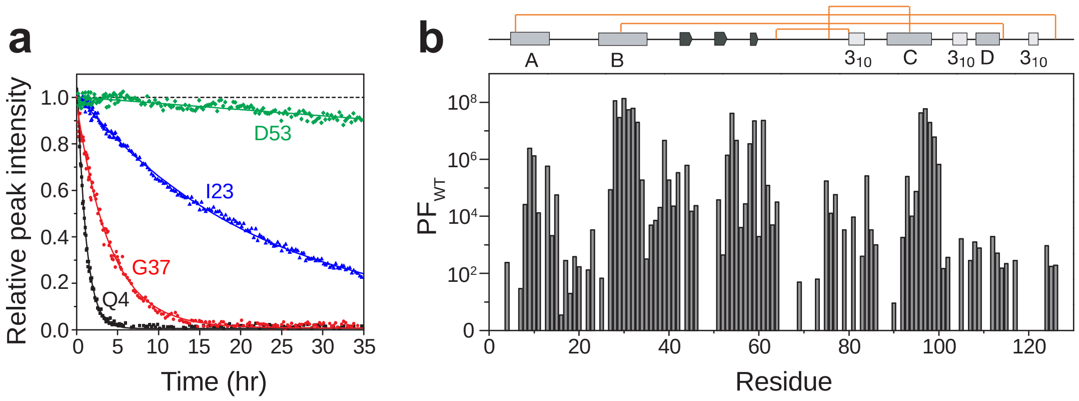

Figure S3. Real-time EX2 HD exchange of the WT protein monitored by NMR. (a) Fitting of peak intensities of four different residues of the WT protein from HSQC spectra to a single exponential decay curve to calculate k_{obs}. (b) Protection factors (PFs) for the amide hydrogen atoms in the WT protein, calculated from the rates of hydrogen exchange monitored by NMR. Residues for which the hydrogen exchange rates are too fast to be measured are left blank.

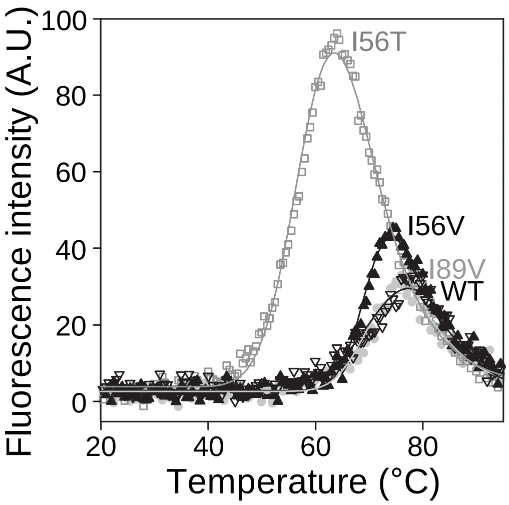

Figure S4. Thermal unfolding of I56V (black, solid triangles) and I89V (light grey circles) monitored by ANS flurosecence emission as in Figure 2(b). All curves were normalised with respect to the maximum fluorescence value of the I56T (dark grey, open squares) variant. Data for I56T and WT (black, open triangles) are shown for comparison.

	$\Delta G^{\circ}(H_2O)_{NU}$ (kJ mol^{-1})	*m* (kJ mol^{-1} M^{-1})	C_m (M)
WT	66.4 ± 3.0 [a]	17.2 ± 0.5 [a]	3.9 ± 0.1 [a]
I59T	39.3 ± 3.3 [a]	13.1 ± 0.7 [a]	3.0 ± 0.1 [a]
I23A	40.6 ± 3.4	13.9 ± 1.1	2.9 ± 0.1

Table S1. Thermodynamic parameters determined from GdnHCl-induced unfolding. [a]Data from (19).
