# Supplementary material for: The Significance of the Location of Mutations for the Native-State Dynamics of Human Lysozyme
Source: Biophys J. 2016 Dec 6;111(11):2358–67. doi: 10.1016/j.bpj.2016.10.028 (PMC5153563; doi:10.1016/j.bpj.2016.10.028)
Supplement: Document S2. Article plus Supporting Material [file mmc2.pdf]

Biophysical Journal
Article

Biophysical Society

The Significance of the Location of Mutations for the Native-State Dynamics of Human Lysozyme

Minkoo Ahn,[1] Christine L. Hagan,[1] Ana Bernardo-Gancedo,[1] Erwin De Genst,[1] Francisco N. Newby,[1] John Christodoulou,[2] Anne Dhulesia,[1] Mireille Dumoulin,[3] Carol V. Robinson,[4] Christopher M. Dobson,[1] and Janet R. Kumita[1,*]

[1]Department of Chemistry, University of Cambridge, Cambridge, United Kingdom; [2]Institute of Structural and Molecular Biology, University College London and Birkbeck College, London, United Kingdom; [3]Laboratory of Enzymology and Protein Folding, Centre for Protein Engineering, InBios, Institute of Chemistry, University of Liege, Liege (Sart Tilman), Belgium; and [4]Physical and Theoretical Chemistry Laboratory, University of Oxford, Oxford, United Kingdom

ABSTRACT The conversion of human lysozyme into amyloid fibrils is associated with a rare but fatal hereditary form of nonneuropathic systemic amyloidosis. The accumulation of large amounts of aggregated protein is thought to be initiated by the formation of transient intermediate species of disease-related lysozyme variants, essentially due to the loss of global cooperativity under physiologically relevant conditions. Interestingly, all five naturally occurring, amyloidogenic, single-point mutations are located in the β-domain of lysozyme, the region that is predominantly unfolded during the formation of the transient intermediate species. Given the lack of known naturally occurring, amyloidogenic, single-point mutations in the α-domain, we chose three specific mutations to address the effects that location may have on native-state dynamics, as studied by hydrogen-deuterium (HD) exchange experiments analyzed by NMR spectroscopy, and mass spectrometry. We compared the effect of a destabilizing α-domain mutation (I23A) with that of the well-characterized I59T β-domain variant. We also investigated the effect of a mutation that has minor effects on native-state stability at the domain interface (I56V) and compared it with that of a variant with similar stability within the C-helix (I89V). We show that when variants have similar reduced native-state stabilities, the location of the mutation (I23A versus I59T) is crucial to the native-state dynamics, with the α-domain mutation having a significantly lower ability to populate transient intermediate species under physiologically relevant conditions. Interestingly, the mutation at the interface (I56V) has a greater effect in facilitating the formation of transient intermediate species at elevated temperatures compared with the variants containing α-domain mutations, even though this mutation results in only minor changes to the native-state stability of lysozyme. These findings reveal that the location of specific mutations is an important factor in determining the native-state dynamical properties of human lysozyme in the context of its propensity to populate the aggregation-prone transient intermediate species associated with pathogenic amyloid formation.

INTRODUCTION

Human lysozyme is a globular protein that acts as a glycosidase and is found in a wide variety of biological fluids (1). This protein, which is stabilized by four disulphide bonds, has two structural domains: the α-domain (residues 1–38 and 86–130), which consists of four α-helices, and the β-domain (residues 39–85), which contains a significant degree of β-sheet structure (2). Lysozyme has been the subject of numerous studies related to its structure, function, folding behavior, and dynamical properties (3). In 1993, two single-point mutations in human lysozyme (I56T and D67H) were found to trigger the manifestation of hereditary nonneuropathic systemic amyloidosis, a rare but fatal disease (4) that is primarily caused by large amounts of amyloid deposits accumulating in the liver, kidneys, and spleen of affected individuals (5,6). Since the discovery of these variants, other naturally occurring disease-associated variants (Y54N, F57I, W64R, F57I/T70N, and T70N/W112R) have been identified, along with two variants (T70N and W112R) that are not linked to disease (7–11).

A range of studies have investigated the effects of these disease-associated mutations on the in vitro folding and misfolding of lysozyme, as well as on amyloid formation. Relative to wild-type (WT) human lysozyme, the amyloidogenic variants I56T and D67H possess reduced native-state stabilities and lower degrees of global structural cooperativity (12–17). Consequently, they are able to populate transient intermediate species in vitro under physiologically relevant

Submitted May 19, 2016, and accepted for publication October 19, 2016.

*Correspondence: jrk38@cam.ac.uk

Editor: Jane Dyson.

http://dx.doi.org/10.1016/j.bpj.2016.10.028

© 2016 Biophysical Society.

This is an open access article under the CC BY license (http://creativecommons.org/licenses/by/4.0/).

CrossMark

conditions. In these intermediate species, the β-domain and the C-helix are cooperatively and significantly unfolded, whereas the remaining parts of the protein maintain a native-like structure (13,15,18). The transient intermediate species can also be detected under similar conditions in the nonnatural variant, I59T, which has a native-state stability lying between that of the I56T variant and WT. Interestingly, the transient intermediate species can be detected in both the T70N variant and WT, but only under more destabilizing conditions (47°C and 57°C, respectively) and not under conditions that are physiologically relevant (19,20).

Previous studies revealed the formation of a transient intermediate species as a crucial step in lysozyme aggregation by examining the binding effects of camelid antibody fragments, often called nanobodies (15,21–23). These studies identified nanobodies that could bind to the native state of the lysozyme variants and prevent fibril formation by suppressing the formation of the transient intermediate species (21,22), or could prevent aggregation at a later stage by inhibiting the unfolding and structural reorganization of the α-domain (23). Further studies under strongly acidic conditions revealed that I56T, I59T, and WT lysozymes unfold via a cooperative loss of native tertiary structure followed by a progressive unfolding of secondary structure, where the β-domain unfolds followed by the C-helix, the first two 3_{10} helices, and some residues at the chain termini (18). Under the same acidic conditions, the relationship between the population of nonnative conformations and amyloid formation rates was established: the higher the population of the ensemble of denatured species, the faster the rate of amyloid formation (24). It was also shown that residues within the β-domain and C-helix form the core of amyloid fibrils when the protein aggregates (25).

Studies of the I56T and D67H proteins have provided detailed information about the structural effects of these mutations. Overall, the structures of the I56T and D67H variants, as determined by x-ray crystallography, resemble that of the WT (12,13). The crystal structure of the I56T protein is almost identical ($RMSD_{C\alpha} < 1$ Å) to that of the WT, whereas the structure of the D67H protein shows larger differences ($RMSD_{C\alpha}$ up to 11 Å) in the residues of the short (residues 45–51) and long (residues 68–75) loops of the β-domain, showing that the two loops are significantly perturbed from their original positions (13). The prominent structural changes in the D67H protein are associated with disruption of one of the two hydrogen-bonding networks that stabilize the β-domain in the WT (2,13). This destabilizing effect is transmitted to I56, resulting in its side chain adopting an alternative conformation (13). I56 is located at the interface between the α- and β-domains, a region that is crucial for the structural integrity of the protein. Thus, it is reasonable to conclude that the amyloidogenic nature of both the I56T and D67H variants is due to the destabilization of the interface region between the two domains of lysozyme (13).

All of the identified disease-associated single-point mutations are significant amino acid changes found in the β-domain of lysozyme, either at the domain interface or in areas with a network of interactions that influences this region. In this study, we set out to examine how the introduction of alternative mutations would modify the properties of lysozyme, and for this purpose we selected previously reported, well-expressed lysozyme variants (I56V and I89V (26), I23A (27), and I59T (16,19,28)). The I23A variant was chosen because this α-domain mutation affects the native-state stability of the protein to a similar degree as the well-characterized I59T (β-domain) variant (19), allowing us to directly compare the effect of a mutation in the α-domain relative to one in the β-domain. We also chose a mutation that does not significantly destabilize the native state of lysozyme but is located in the critical domain interface region, I56V. For comparison with this variant, we selected the I89V mutation, which has a comparable native-state stability and is located in the C-helix region (Fig. 1 *a*). We used real-time and pulse-labeling hydrogen deuterium (HD) exchange experiments, monitored by NMR and mass spectrometry (MS), respectively, and compared the results with previous findings for the I56T, I59T, D67H, and T70N variants. The results indicate that the site-specific location of a mutation can significantly contribute to altering the native-state dynamics in variants with mutations that are not greatly destabilizing. Therefore, in combination, the location of the amyloidogenic mutation and the reduced native-state stability are key elements in lysozyme fibril formation.

MATERIALS AND METHODS

All chemicals were purchased from Sigma-Aldrich (Gillingham, UK) unless otherwise stated.

Protein expression and purification

Lysozyme variants were expressed in *Pichia pastoris* and purified as described previously (20). Protein purity (>95%) was based on SDS-PAGE and the molecular masses were confirmed by MS.

Thermal denaturation measured by circular dichroism and fluorescence spectroscopies

Circular dichroism (CD) spectroscopy experiments were performed in a Jasco J-810 spectropolarimeter (JASCO, Great Dunmow, UK) equipped with a Peltier temperature controller. The proteins (20 μM) were dissolved in sodium citrate buffer (10 mM, pH 5) and analyzed in a 0.1 or 1 cm cuvette. Thermal denaturation was monitored at 222 or 270 nm with temperature increasing from 5°C to 95°C (0.5°C min^{-1}). Ellipticity values were normalized to the fraction of unfolded protein (F_u) using

$$Fu = \frac{(\theta - \theta_N)}{(\theta_U - \theta_N)},$$

where θ is the observed ellipticity, and θ_N and θ_U are the native- and unfolded-state ellipticities, respectively. θ_N and θ_U were extrapolated

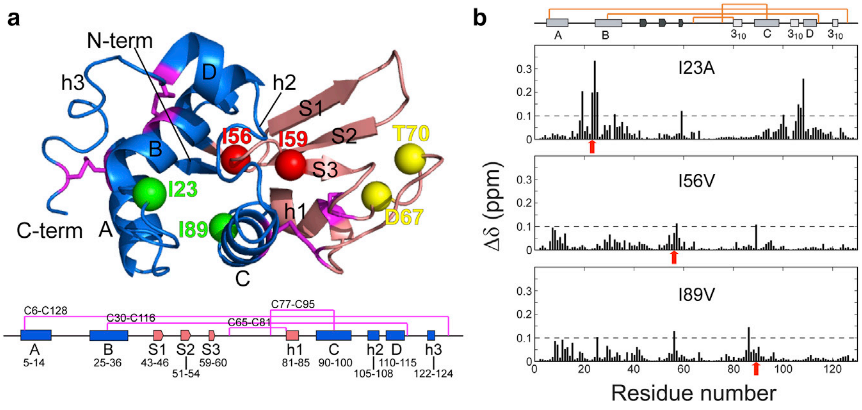

FIGURE 1 Human lysozyme mutations discussed in this study. (*a*) Mutations in the α-domain (I23A and I89V) (*green*), the β-domain (D67H and T70N) (*yellow*), and the interface region (I56T, I56V, and I59T) (*red*). The location of the α-domain α-helices (A, B, C, and D), β-strands in the β-domain (S1, S2, and S3), and 3_{10} helices (h1, h2, and h3) are shown below the structure, with magenta lines representing disulphide bonds. (*b*) Chemical-shift perturbations in the NMR spectra (pH 5.0, 37°C) of the I23A, I56V, and I89V variants. Chemical-shift changes are defined as $\Delta\delta = ((\Delta\delta^1\mathrm{H})^2 + (\omega\Delta\delta^{15}\mathrm{N})^2)^{0.5}$, where $\omega = \gamma_{15N}/\gamma_{1H}$ (19,38), and the threshold for significant chemical-shift differences was fixed at 0.1 ppm (19,21). Red arrows indicate the sites of mutation.

from pre- and posttransition baselines at the relevant temperature. Data were fitted to a two-state unfolding model (18,29) using OriginPro 8.0 (OriginLab, Northampton, MA). Mid-point T_m values are defined as the temperatures at which $F_u = 0.5$. Thermal denaturation monitored by 8-anilino-1-naphthalenesulfonic acid (ANS) fluorescence emission was recorded on a Cary Eclipse spectrofluorimeter (Agilent, Oxford, UK) using excitation/emission wavelengths of 350 and 475 nm, respectively (slit widths: 5 nm) with increasing temperatures from 20°C to 95°C (0.5°C min^{-1}). Samples contained 2 μM protein (0.1 M sodium citrate pH 5.0, 360 μM ANS). The transition curves of ANS alone were collected and subtracted from all samples. The fluorescence data were normalized with respect to the ANS emission signal in the presence of the I56T variant. Data were fitted to a Gaussian expression using OriginPro 8.0.

Chemical denaturation monitored by intrinsic fluorescence

The WT, I23A, and I59T proteins were incubated (50 mM sodium phosphate pH 6.5, 25°C, 18 h) in the presence of increasing guanidinium hydrochloride (GdnHCl) concentrations, with protein concentrations of 1.6, 2.1, and 2.1 μM, respectively. The reversibility of the unfolding transitions was checked by diluting the samples from post-transition (6 M) to pre-transition GdnHCl concentrations. Unfolding and refolding curves were determined by monitoring the intrinsic fluorescence emission. Each GdnHCl concentration was determined by refractometry (30). Fluorescence spectra were recorded on a LS55 Luminescence spectrometer (Perkin Elmer, Seer Green, UK) in a 1 cm quartz mini-cell. The spectra were recorded in order of increasing GdnHCl concentration. Samples were excited at 295 nm, emission spectra were collected (310–440 nm) with slit widths of 5 nm, and scans were collected at 60 nm/min (25°C, PMT = 900 V). The control sample spectra were subtracted from the corresponding protein spectra. The thermodynamic parameters were computed under the assumption of a two-state model for the unfolding reaction. The corrected fluorescence intensity at 355 nm was plotted against the GdnHCl concentration in each sample, and the data were fit as described in (19) to

$$S = \frac{\alpha_N + \beta_N[D] + (\alpha_U + \beta_U[D])e^{-\left(\frac{\Delta G^\circ(H2O)_{NU} - m[D]}{RT}\right)}}{1 + e^{-\left(\frac{\Delta G^\circ(H2O)_{NU} - m[D]}{RT}\right)}},$$

where S represents the fluorescence signal and $[D]$ represents the GdnHCl concentration. αN and αU define the intercepts, and βN and βU define the slopes of the pre- and post-transition regions, respectively. $\Delta G^\circ(\mathrm{H_2O})_{\mathrm{NU}}$ and m respectively define the difference in free energy between the folded and unfolded conformations in the absence of denaturant and the dependence of the free energy on the denaturant concentration. The midpoint of the denaturation curve is given by $C_m = \Delta G^\circ(\mathrm{H_2O})_{\mathrm{NU}}/m$.

In vitro fibril formation

Aggregation studies were performed in triplicate with lysozyme variants (6.8 μM, 0.1 M sodium citrate buffer, pH 5.0, 62.5 μM thioflavin-T (ThT)) incubated with stirring at 60°C in a Cary Eclipse spectrofluorimeter. ThT fluorescence was measured with excitation and emission wavelengths of 440 nm and 480 nm, respectively (slit widths: 5 nm). Samples for transmission electron microscopy were prepared on carbon support film, 400 mesh, 3 mm copper grids (EM Resolutions, Saffron Walden, UK) and stained with 2% uranyl acetate (w/v). The samples were imaged on an FEI Tecnai G_2 transmission electron microscope (CAIC, University of Cambridge, Cambridge, UK). Images were analyzed using the SIS Megaview II Image Capture system (Olympus, Tokyo, Japan).

HD exchange by electrospray ionization MS

EX1 HD exchange of variants was monitored by electrospray ionization MS (pH 8, 37°C or 47°C) and the data were processed as previously described (19,20). Proteins were deuterated at exchangeable sites by dissolving lysozyme (0.2 mg) in D_2O (pH 3.8, 350 μL), followed by heating (70°C, 10 min) and then cooling to room temperature. The samples were then lyophilized. Before HD exchange, the samples were dissolved in D_2O (200 μM) and HD exchange was triggered by manual mixing as previously described (20). Spectra were acquired over a mass/charge range of 500–5000 Da on an LCT MS instrument (Waters, Elstree, UK) equipped with a nanoflow Z-spray source and calibrated with cesium iodide (15 μM). Data were analyzed using MassLynx 4.0 (Waters) with molecular masses calculated from the centroid values of at least three charge states. The mass spectra represent the convolution of three charged species with minimal smoothing and conversion to a mass scale. The relative intensity of the intermediate (lower-mass) species to the native-state protein (higher-mass species) was plotted against the pulse labeling time. The data were fit to a single exponential curve and the unfolding rate (k) was calculated. The time constant of the unfolding process is the reciprocal of the unfolding rate ($\tau = 1/k$). All reported τ-values are the average of three to five separate experiments.

Real-time HD exchange by NMR

^{15}N-labeled lysozyme variant solutions were prepared in a standard 3 mm NMR tube (2 mg, 220 μL of 20 mM D-4 acetic acid in D_2O (pD 5)), as previously described (17,20). The samples were immediately placed in a

Bruker Avance 500 MHz NMR spectrometer (Bruker, Coventry, UK), pretuned, and shimmed with an equivalent sample at 37°C. A series of heteronuclear single quantum coherence (HSQC) spectra were recorded with two (first 30 spectra), four (next 30 spectra), and eight (all subsequent spectra) transients, with 1792 and 96 complex points in *t*2 and *t*1, respectively, and sweep widths of 6010 and 1014 Hz in F2 and F1, respectively. The observed HD exchange rates (k_{obs}) were measured from peak height reductions over time by fitting the data to a single exponential decay with or without a constant as a baseline. The predicted rates (k_{ex}) for a random coil model based on the primary structure were calculated by Sphere (http://www.fccc.edu/research/labs/roder/sphere/) using the pH values of the samples measured at the end of the experiments. Protection factors (PFs) were calculated from the ratio of k_{obs} to k_{ex}. The ratio of the PF values was calculated by dividing the PFs of WT by those of each variant.

RESULTS

The I23A, I56V, and I89V mutations do not significantly alter the lysozyme native-state solution structure

To understand the effects of the I23A, I56V, and I89V mutations on the native-state structure of lysozyme, we recorded HSQC-NMR spectra and compared them with that obtained for the WT. The spectra for all variants closely overlaid the WT spectrum, with only minor changes in a small number of crosspeaks. The backbone amide chemical shifts were calculated for each variant and confirmed the absence of major perturbations (Fig. 1 *b*; Fig. S1 in the Supporting Material). Thus, the global folds of the native-state structure of the three variants are effectively the same.

Almost all chemical-shift differences ($\Delta\delta$ values) between the I56V or I89V variants and the WT are lower than the threshold (0.1 ppm), again confirming the structural similarity between the proteins. There are, however, small but reproducible differences, including F57 and I89 in the I56V variant and I56 and I86 in the I89V variant, all of which are located at the domain interface region (residues 54–59) or in the loop between the first 3_{10} helix (h1) and the C-helix (residues 86–89), which are in close spatial proximity to the interface (26). Compared with the I56V and I89V variants, the I23A protein has a larger number of affected residues and shows more chemical-shift differences compared with the WT. The residues that show significant $\Delta\delta$ values are all located near the mutation site in the α-domain, either sequentially (residues 19–25) or spatially (residues 98–109) (Fig. 1 *b*), and are involved in interactions within the hydrophobic core where residue I23 is located (26). Interestingly, in the I23A variant, residue I59, located at the domain interface, also shows significant chemical-shift perturbations, though this residue is not in close proximity to the mutation site (27). Considering the fact that I56V and I89V also exhibit noticeable chemical-shift perturbations in this region, it appears that the interface region is sensitive to small structural changes within the protein.

Taken together, the results show that the three mutations do not significantly alter the overall native-state structure of lysozyme. The slightly higher chemical-shift perturbations observed in I23A may be explained by a larger cavity in the hydrophobic core of the α-domain created by the Ile-to-Ala mutation (27). In the absence of internal water molecules, the cavity in I23A, which is surrounded by the B-, C-, and D-helices, is likely to undergo subtle changes in the microscopic environment and hence generate small chemical-shift perturbations.

The I23A mutation destabilizes the native state of lysozyme

Here, we assessed the native-state stability of the I23A variant, as well as the relative stability of all variants, as compared with the WT using circular dichroism (CD) spectroscopy (15,16,19) (Table 1). The resulting data are in good agreement with the differential scanning calorimetry experiments reported by Takano et al. (26,27) and Funahashi et al. (28). At pH 5.0, the I89V and I56V variants show a native-state stability with comparable T_m values. As previously reported, the pathogenic variant, I56T, has the lowest native-state stability, and the stabilities of T70N, a natural, nonamyloidogenic variant, and I59T, a nonnatural variant, lie between the native-state stabilities of the I56T variant and WT.

Unlike I56V and I89V, which do not have large effects on lysozyme native-state stability, I23A has a significant destabilizing effect, displaying a T_m comparable to that of the I59T variant, which possesses amyloidogenic characteristics under physiologically relevant conditions (Fig. 2 *a*) (19). The I23A variant shows an increase in ANS fluorescence emission at temperatures close to its T_m due to the appearance of partially unfolded intermediates with solvent-exposed hydrophobic clusters, and the intensity of the ANS fluorescence is comparable to that of the I59T variant (Fig. 2 *b*). GdnHCl-induced unfolding of the I23A variant using intrinsic fluorescence allowed us to determine that the $\Delta G°(H_2O)_{NU}$ value for I23A is 40.6 ± 3.4 kJ mol^{-1}, which is effectively the

TABLE 1 T_m **Values of Lysozyme Variants Measured by Thermal Denaturation Monitored by Far-UV and Near-UV CD Spectroscopy at pH 5.0**

	T_m (°C)	
Lysozyme Variant	Far-UV	Near-UV
WT	77.7 ± 0.5[a]	76.7 ± 0.6[b]
I89V	77.1 ± 1.0	77.2 ± 0.5
I56V	76.2 ± 0.9	75.3 ± 0.6
T70N	74.0 ± 0.6[a]	72.2 ± 1.0[c]
I59T	71.2 ± 0.4[a]	69.9 ± 0.4[b]
I23A	69.8 ± 1.0	69.7 ± 1.2
I56T	67.6 ± 0.8[a]	66.2 ± 1.0[b]

Values are an average of three experiments with the reported standard deviations.
[a]Data from (16).
[b]Data from (19).
[c]Data from (20).

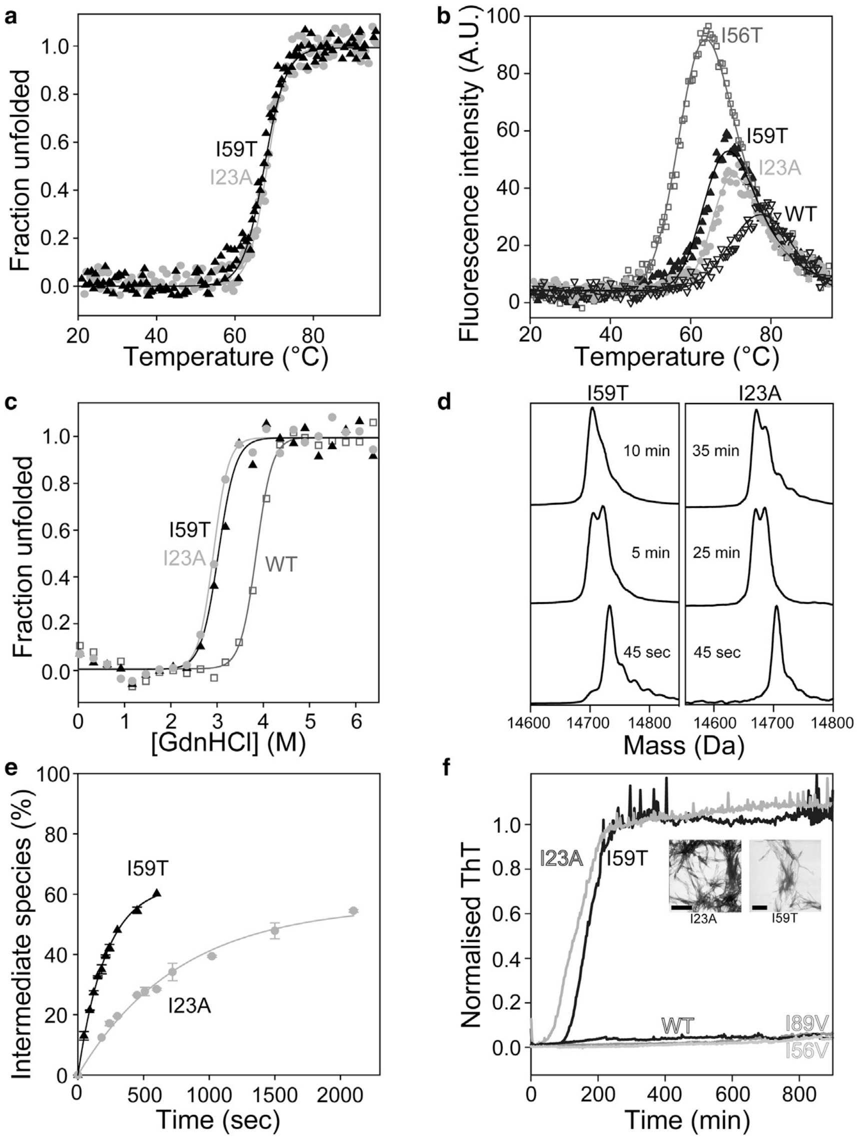

FIGURE 2 Biophysical characteristics of the I23A and I59T variants. (*a*) Thermal denaturation of I23A (*gray circles*) and I59T (*black triangles*) monitored by far-UV CD (222 nm). (*b*) Partially unfolded intermediates observed by ANS fluorescence emission. Curves are representative data sets normalized with respect to the maximum fluorescence of the I56T variant (*dark gray open squares*). I23A (*light gray circles*) and I59T (*black solid triangles*) show similar degrees of ANS signal. The peak observed for the WT (*black open triangles*) shows the lowest intensity. The T_{mANS} values for I56T, I23A, I59T, and WT are 65.6°C ± 0.8°C, 72.8°C ± 1.3°C, 71.0°C ± 0.7°C, and 79.2°C ± 1.4°C, respectively. (*c*) GdnHCl-induced unfolding of I23A (*light gray circles*), I59T (*black triangles*), and WT (*dark gray open squares*) proteins monitored with intrinsic fluorescence measurements. (*d*) HD exchange monitored by MS for I23A and I59T (pH 8, 37°C). Initially, the proteins show a prominent peak corresponding to the deuterated native protein (higher-mass species) and as time progresses, a second, lower-mass peak appears, where deuteron-proton exchange has occurred in relation to the formation of the transient, partially unfolded intermediate species. (*e*) Plot of the time-dependent increase in the intermediate (lower-mass) species population of I23A (*gray circles*) and I59T (*black triangles*) at 37°C. (*f*) Aggregation of lysozyme variants monitored at pH 5.0, 60°C, with stirring shows that I23A ($t_{1/2}$ = 140 ± 50 min) and I59T ($t_{1/2}$ = 170 ± 40 min) formed ThT-positive fibrils, whereas I56V, I89V, and WT did not. Inset: transmission electron microscopy images of the I23A and I59T fibrils (scale bars, 500 nm).

same as the value obtained for I59T (39.3 ± 3.3 kJ mol^{-1}; Fig. 2 *c*; Table S1).

Lysozyme native-state dynamics are affected by the location of the mutation

The comparable native-state stabilities of the I23A and I59T variants provided us an opportunity to compare how mutations in different domains affect the population of the transient intermediate species relevant to fibril formation. We monitored HD exchange using MS at pH 8, where the exchange is primarily in the EX1 regime due to the high exchange rate (k_{ex}) relative to the closing rate (k_{cl}), and which has previously allowed the observation of transient intermediate states in the I56T and D67H variants (13,14,17,21). Specifically, we observed bimodal peak distributions: one representing a population of molecules that never access the partially unfolded species, and one representing a population that accesses transiently, at least once, the intermediate species via locally cooperative unfolding events. The relative intensities of the lower-mass species at different times were fitted to a single-exponential curve to obtain the unfolding rate (k), from which the time constant of the unfolding process ($\tau = 1/k$) could be determined. It has been shown that the I59T variant populates the intermediate species under the same physiologically relevant conditions (37°C and pH 8) as do the naturally occurring amyloidogenic variants, albeit at a slower rate (τ = 30.0 and 212.8 s for I56T and I59T, respectively) (19). When we compared I23A and I59T, we observed that, despite the similarity in native-state stability, the I23A variant populated the transient, partly unfolded species with a time constant of unfolding nearly four times larger than that of I59T (769.2 and 212.8 s, respectively; Fig. 2,

d and *e*; Table 2). This is much larger than the time constant of unfolding observed under similar conditions for the I56T and D67H variants (30.0 and 15.3 s, respectively). We compared the number of protected sites within the higher-mass and lower-mass peaks for I23A and I59T (Fig. S2), and found the same degree of protection for both proteins, confirming that both variants have a similar number of sites that are not accessible to DH solvent exchange and therefore are not dramatically different in overall structure. It appears that the location of the destabilizing mutation plays a role in how the protein populates the transient intermediate species.

To compare the HD exchange behaviors of the more stable variants, I56V and I89V, we performed the experiments at 47°C, since at this temperature intermediate species could be detected for the T70N variant (20). At this elevated temperature, intermediate species were transiently formed for all variants, with the rate of formation being much faster than at 37°C, and the time constant of unfolding was still threefold higher for I23A compared with I59T (Table 2; Fig. 3 *a*). Interestingly, when we examined the I56V, I89V, and T70N variants, we found that the ability to populate the transient intermediate species was greater for the I56V and T70N proteins, whose time constants of unfolding were similar to that of I59T under the same conditions, whereas I89V showed the slowest rate of intermediate formation, with a sixfold larger time constant of unfolding compared with I59T (Table 2; Fig. 3 *b*). Although the I56V and I89V mutations had similar effects on lysozyme thermostability, the mutation at the interface region had a more pronounced effect on the global cooperativity of lysozyme and on the formation of the transient intermediate species under destabilizing conditions.

The effects of different mutations on the native-state dynamics were examined on a residue-specific level using HD exchange monitored by NMR. At pH 5 and 37°C, the exchange rate is in the EX2 regime, where the closing rate (k_{cl}) is much faster than the intrinsic exchange rate (k_{ex}) and the latter step becomes rate limiting, thereby providing information about the native-state fluctuations of the protein (15,17,19,20). HD exchange measurements for the variants were performed and the ratios of the WT PFs to those of each variant were determined. A decrease in PFs for the variant proteins leads to an increase in this ratio, and this is considered significant when the ratio is higher than 10, indicating an increase in native-state dynamics (Fig. 4).

Under these conditions, the I56T and D67H variants show dramatically reduced PFs relative to the WT, primarily for the residues in the β-domain and the C-helix (15,17). The I59T and T70N variants, which are more stable than I56T and D67H, show smaller decreases in the PFs, particularly in the β-domain region (19,20). The reduced PFs in the β-domain of I59T and T70N, as well as the C-helix of I59T, reveal that they are more accessible to the solvent, an effect that is attributable to the reductions in global cooperativity generated by these mutations. The EX2 exchange kinetics of the I23A, I56V, and I89V variants were compared with those of the I59T, T70N, and WT proteins. The PFs obtained for the WT show the same trend and values as reported previously (Fig. S3) (17), with residues involved in secondary-structure elements being highly protected from exchange except in the 3_{10}- and D-helices, which are highly exposed to the solvent. In I59T, dramatic decreases in the PFs are observed predominantly in a region of the β-sheet (residues 50–65), with smaller changes detected in the C-helix (residues 90–100; Fig. 4 *a*). In contrast, T70N shows smaller PF ratios than I59T, but in a more extended region of the β-sheet (residues 38–86; Fig. 4 *b*). Interestingly, the I56V variant shows significant differences in the PF ratios of some residues in the β-sheet region (residues 50–65; Fig. 4 *c*), although the decreases in the PFs of I56V are smaller than those of I59T. Nevertheless, the small cavity created by the I56V mutation at the domain interface appears to affect the protein dynamics in a manner similar to that observed for the amyloidogenic variants. I23A only shows significant changes in PF ratios in the B-helix and does not affect residues located in the β-domain or the C-helix (Fig. 4 *d*). This behavior is clearly very different from that of the I59T, T70N, and I56V proteins, as well as the pathogenic variants. Combined with the slower rate of the unfolding process observed in the EX1 HD exchange experiments, this may suggest possible differences in the mechanism by which I23A is able to populate the transient intermediate species, or in the structural nature of the intermediate species populated by I23A relative to the I59T variant. Finally, the HD exchange results for the I89V variant show no change from the WT (Fig. 4 *e*).

TABLE 2 Time Constants of the Unfolding Process ($\tau = 1/k$) for Variants at 37°C and 47°C

Lysozyme Variant	T_m (°C)	τ (s) 37°C	τ (s) 47°C
I89V	77.1 ± 1.0	N/D	66.7 ± 8.9
I56V	76.2 ± 0.9	N/D	13.5 ± 0.5
T70N	74 ± 0.6	N/D	8.6 ± 2.0
I59T	71.2 ± 0.4	212.8 ± 4.5	11.6 ± 0.9
I23A	69.8 ± 1.0	769.2 ± 4.5	34.5 ± 6.0
I56T	67.6 ± 0.8	30.0 ± 2.5[a]	N/A
D67H	68.0 ± 1.0[b]	15.3 ± 0.6[b]	N/A

A bimodal distribution is not detected (N/D) for I89V, I56V, and T70N at 37°C. The rates of intermediate formation for I56T and D67H are too fast to monitor at 47°C (under the current experimental setup) and are not available (N/A).
[a]Data from (19).
[b]Data from (15).

In vitro aggregation of lysozyme variants

Given that we observed significant differences in native-state dynamics between the I23A and I59T variants, and also between the I56V and I89V variants, we investigated

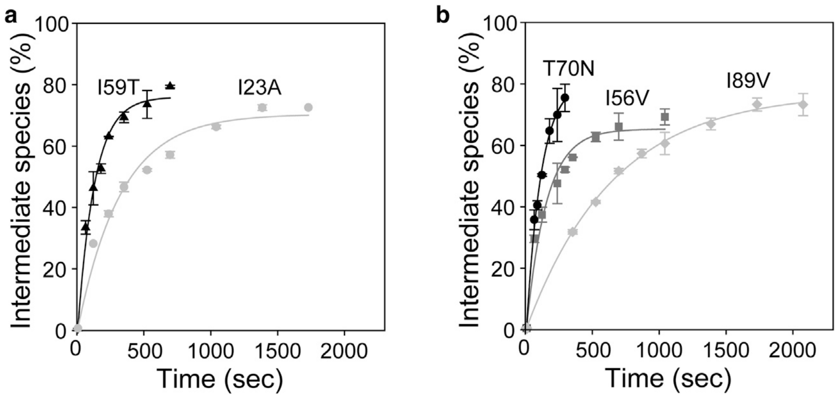

FIGURE 3 EX1 HD exchange monitored by MS for lysozyme variants (pH 8, 47°C). (*a*) Time-dependent increase in the intermediate species population for I23A (*light gray squares*) and I59T (*black triangles*). (*b*) Time-dependent increase in the intermediate species population for T70N (*black circles*), I56V (*gray squares*), and I89V (*light gray circles*).

fibril formation by these variants. Lysozyme is an extremely stable protein, and numerous aggregation conditions have been reported, including low pH and high temperatures (18), high concentrations and low pH (31), and denaturants at high temperatures (21). For I59T lysozyme, we previously employed aggregation conditions using a high temperature (60°C) at pH 5.0 and with stirring. These conditions did not result in fibril formation by T70N or WT, but I59T

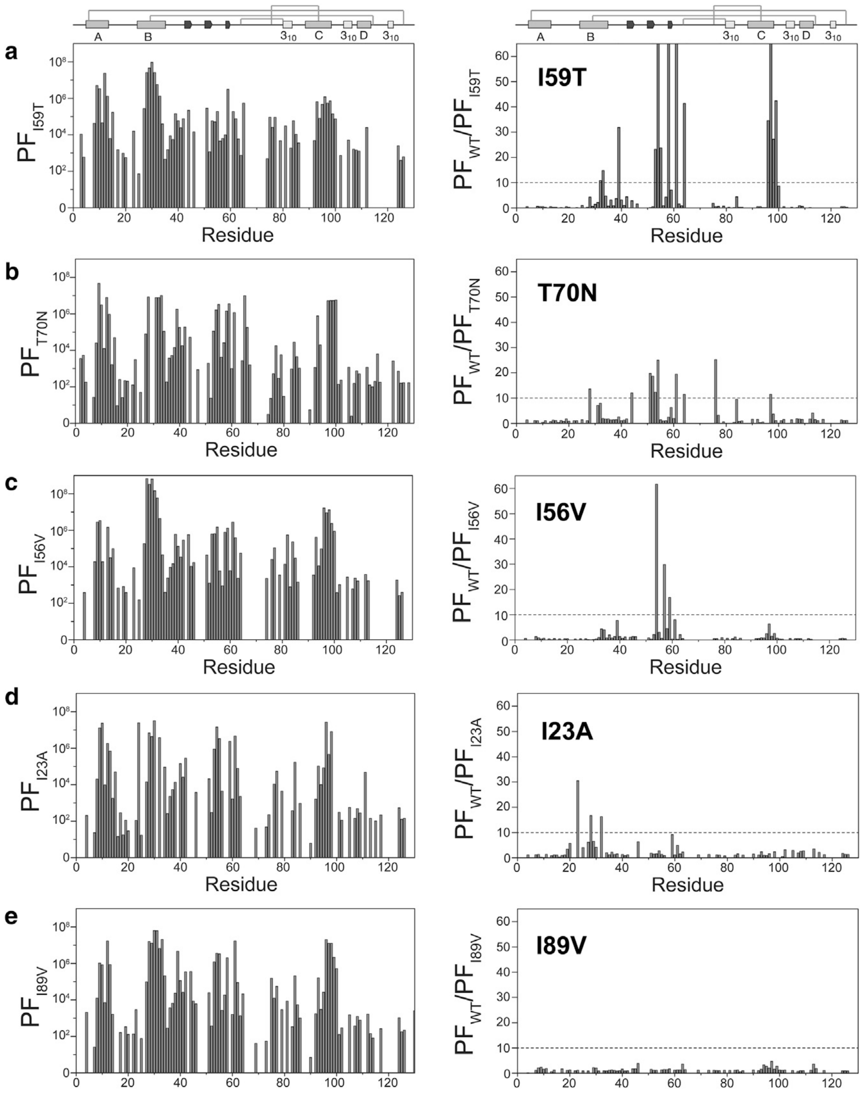

FIGURE 4 (*a–e*) PFs and WT/variant PF ratios for (*a*) I59T, (*b*) T70N, (*c*) I56V, (*d*) I23A, and (*e*) I89V measured by NMR (pH 5, 37°C). For some residues, a lack of corresponding PF data for both the variant and WT leads to an absence of calculated WT/variant PF ratios.

and I56T were found to form fibrils (19). When we examined the fibril formation of I23A, I59T, I56V, I89V, and WT at pH 5.0, 60°C, over a time course of 900 min, only I23A and I59T formed mature fibrils, and no significant differences in the rates of fibril formation were observed for these variants despite the differences in their native-state dynamics (Fig. 2 *f*). The relatively harsh aggregation conditions needed to produce in vitro lysozyme fibrils suggest that the relative rates of aggregation in these conditions correlate with the native-state stabilities of the proteins.

DISCUSSION

Despite their stable natively folded structure under physiological conditions, a range of globular proteins undergo aggregation and cause protein deposition diseases (32,33). In general, a process of global, or at least partial, unfolding of the protein is required to initiate aggregation, eventually giving rise to amyloid formation (34,35). In most cases, transient and partial unfolding of the protein creates conformational states that are thermodynamically close to the native state (32). Human lysozyme, consistent with this behavior, forms transiently populated intermediate states that appear to be the precursors of the amyloid fibrils associated with hereditary, nonneuropathic, systemic amyloidosis (13,17,21,36). Extensive studies on human lysozyme have reported that the pathogenic variants show decreases in native-state stability and global cooperativity, and these attributes were linked to an increase in the formation of partially unfolded, transient intermediate species (13,15,17). As all of the known natural single-point disease-associated mutations in lysozyme are in the β-domain region, we examined the native-state properties and dynamics of three nonnatural lysozyme variants with mutations in different locations: an α-domain mutation that significantly decreases native-state stability, and a mutation in the interface region and a mutation in the α-domain, both of which do not result in a large decrease in native-state stability.

The I23A variant has a significantly lower thermostability than the I56V and I89V variants (Table 1). Indeed, the I23A variant has a native-state stability similar to that of the I59T variant (Fig. 2; Table 2) (19). In contrast to the I59T variant, the EX2 HD exchange pattern of I23A did not show dramatic effects of the mutation on the native-state dynamics within the β-domain and C-helix regions; only slight increases were observed in a small number of residues near the mutation site. Differences were also evident when the EX1 HD exchange was monitored by MS, which showed that although the I23A variant is capable of forming transient intermediate species under physiologically relevant conditions, the time constant of unfolding is nearly fourfold greater than that observed for the I59T variant. This suggests that the location of the mutation can alter how the protein populates the transient intermediate species, and may also cause changes in the structural nature of this species. When this variant was compared with others at 47°C, it showed a much greater time constant of unfolding compared with the I59T, T70N, and I56V proteins. Despite its great importance, the reduced native-state stability of the protein is not the only determining factor in populating the transient intermediate state of lysozyme.

The effect of the I56V mutation on the lysozyme native-state dynamics is very significant, despite the fact that it shows little difference in native-state stability compared with the I89V and WT proteins. A comparison of I56V and I89V in ANS denaturation experiments shows that I56V displays a higher ANS fluorescence than the I89V and WT proteins, with a maximum fluorescence similar to that of the I23A variant (Fig. S4). The I56V variant displays a trend in local fluctuations similar to that observed for I59T and other pathogenic lysozymes: the PFs of the β-sheet residues are greatly decreased, whereas those in other parts of the protein remain unchanged. Despite this similar trend, no transient intermediate is observed for I56V at 37°C, although at 47°C the I56V variant populates the transient intermediate species with a time constant of unfolding similar to that of the I59T and T70N variants (Table 2), and the I89V variant, although similar in thermostability to I56V, has a much slower rate of intermediate formation at 47°C.

Overall, a comparison of the effects of mutations at three different locations on lysozyme native-state stability and dynamics reveals the importance of the mutations' location with regard to population of the transient intermediate species. Mutations in the B- or C-helix of the α-domain have a significantly smaller effect on the ability to populate the intermediate species regardless of their overall effect on the lysozyme native-state stability, whereas even conservative mutations made in the interface region can alter the local and global dynamic fluctuations as observed for I56V. As previously reported, I56 has a crucial role in maintaining the structural integrity of the protein, linking the two domains of human lysozyme at their interface (13). Hence, when it is substituted by a polar residue, such as I56T, the hydroxyl group of threonine in the hydrophobic core of the protein causes the disruption of intramolecular hydrogen bonds (12). As a result, the I56T protein significantly loses global cooperativity and shows a drastic decrease in native-state stability. Although the Ile-to-Val mutation in I56V is a more subtle change than Ile-to-Thr, it can still result in significant changes to the native dynamics, which may facilitate the destabilization and release of the small loop at the domain interface to the solvent. Interestingly, despite the fact that the native-state stability of I56V was not greatly perturbed, at 47°C this variant was able to readily populate the transient, partially unfolded intermediate species that are critical for lysozyme aggregation. Given the evidence that the location of point mutations plays a role in whether lysozyme possesses attributes that may facilitate misfolding, it

may not be a coincidence that all five natural single-point mutations greatly affect the small loop region at the domain interface, either as a result of the mutation being within this region (Y54N, I56T, and F57I) or through long-range destabilizing effects transmitted from the long loop in the β-sheet (W64R and D67H). Although we could not observe a relationship between the differences in native-state dynamics and the propensity to form fibrils, this observation likely reflects the fact that nonphysiologically relevant conditions were needed to initiate in vitro lysozyme fibril formation. Therefore, it is very possible that the location of the mutation could be a crucial factor in the propensity for lysozyme aggregation in patients suffering from systemic amyloidosis.

CONCLUSION

By examining specific mutations (I23A, I56V, and I89V) in human lysozyme, we found that the location of the mutation influences native-state stability and dynamics, and the formation of partially unfolded transient intermediate species in the protein. Even though the I23A variant (α-domain) has a similarly reduced native-state stability compared with I59T (β-domain), it results in a significantly lower ability to populate intermediate species under physiologically relevant conditions. Interestingly, I56V, a mutation that does not greatly perturb lysozyme native-state stability, has increased native-state dynamics and at elevated temperatures can readily populate the partially unfolded transient intermediate species. Furthermore, as monitored by NMR, the local fluctuations of I56V are similar to those of I56T and I59T, whereas the I89V variant, which is similar in thermostability to I56V, shows no changes in PFs as compared with the WT. These findings indicate that mutations in specific regions of lysozyme can change the native-state dynamics and how the protein forms partially unfolded intermediate species. In particular, changes within the interface region appear to be critical, consistent with previous reports (37) indicating that the structural integrity of lysozyme hinges on the structure and dynamics of the interface region between the two domains.

SUPPORTING MATERIAL

Four figures and one table are available at http://www.biophysj.org/biophysj/supplemental/S0006-3495(16)30960-2.

AUTHOR CONTRIBUTIONS

M.A., C.L.H., C.M.D., and J.R.K. designed the research. M.A., C.L.H., A.B.-G., E.D.G., F.N.N., A.D., J.C., and J.R.K. performed experiments. M.A., C.L.H., A.B.-G., E.D.G., F.N.N., J.C., A.D., M.D., C.V.R., C.M.D., and J.R.K. analyzed the data. M.A., C.M.D., and J.R.K. wrote the manuscript.

ACKNOWLEDGMENTS

This research was supported by the Biotechnology and Biological Sciences Research Council (BB/E019927/1 to C.M.D., C.V.R., and J.R.K.), the Medical Research Council (E.D.G. and C.M.D.), the Belgian Program of Interuniversity Attraction Poles administered by the Federal Office for Scientific Technical and Cultural Affairs (PAI numbers P6/19 and P7144 to C.M.D. and M.D.), the European Union's Sixth Framework Program (LSHM-CT-2006-037525 to C.M.D. and M.D.), and Programme grants from the Wellcome Trust and the Leverhulme Trust (C.M.D.). It was also supported by a Korean Government Scholarship for Overseas Studies (M.A.), the Winston Churchill Foundation (C.L.H.), and Boerhinger Ingleheim funds (A.D.). The NMR facility at the Department of Chemistry, University of Cambridge, is supported in part by an EPSRC Core Capability grant (EP/K039520/1).

REFERENCES

1. Fleming, A. 1922. On a remarkable bacteriolytic element found in tissues and secretions. *Proc. R. Soc. Lond. B Biol. Sci.* 93:306–317.
2. Artymiuk, P. J., and C. C. Blake. 1981. Refinement of human lysozyme at 1.5 A resolution analysis of non-bonded and hydrogen-bond interactions. *J. Mol. Biol.* 152:737–762.
3. Dumoulin, M., J. R. Kumita, and C. M. Dobson. 2006. Normal and aberrant biological self-assembly: Insights from studies of human lysozyme and its amyloidogenic variants. *Acc. Chem. Res.* 39:603–610.
4. Pepys, M. B., P. N. Hawkins, ..., J. J. Hsuan. 1993. Human lysozyme gene mutations cause hereditary systemic amyloidosis. *Nature.* 362:553–557.
5. Gillmore, J. D., D. R. Booth, ..., P. N. Hawkins. 1999. Hereditary renal amyloidosis associated with variant lysozyme in a large English family. *Nephrol. Dial. Transplant.* 14:2639–2644.
6. Pepys, M. B. 2001. Pathogenesis, diagnosis and treatment of systemic amyloidosis. *Philos. Trans. R. Soc. Lond. B Biol. Sci.* 356:203–210, discussion 210–211.
7. Booth, D. R., M. B. Pepys, and P. N. Hawkins. 2000. A novel variant of human lysozyme (T70N) is common in the normal population. *Hum. Mutat.* 16:180.
8. Valleix, S., S. Drunat, ..., G. Grateau. 2002. Hereditary renal amyloidosis caused by a new variant lysozyme W64R in a French family. *Kidney Int.* 61:907–912.
9. Yazaki, M., S. A. Farrell, and M. D. Benson. 2003. A novel lysozyme mutation Phe57Ile associated with hereditary renal amyloidosis. *Kidney Int.* 63:1652–1657.
10. Röcken, C., K. Becker, ..., F. W. Albert. 2006. ALys amyloidosis caused by compound heterozygosity in exon 2 (Thr70Asn) and exon 4 (Trp112Arg) of the lysozyme gene. *Hum. Mutat.* 27:119–120.
11. Girnius, S., M. Skinner, ..., L. H. Connors. 2012. A new lysozyme tyr54asn mutation causing amyloidosis in a family of Swedish ancestry with gastrointestinal symptoms. *Amyloid.* 19:182–185.
12. Funahashi, J., K. Takano, ..., K. Yutani. 1996. The structure, stability, and folding process of amyloidogenic mutant human lysozyme. *J. Biochem.* 120:1216–1223.
13. Booth, D. R., M. Sunde, ..., M. B. Pepys. 1997. Instability, unfolding and aggregation of human lysozyme variants underlying amyloid fibrillogenesis. *Nature.* 385:787–793.
14. Canet, D., M. Sunde, ..., C. M. Dobson. 1999. Mechanistic studies of the folding of human lysozyme and the origin of amyloidogenic behavior in its disease-related variants. *Biochemistry.* 38:6419–6427.
15. Dumoulin, M., D. Canet, ..., C. M. Dobson. 2005. Reduced global cooperativity is a common feature underlying the amyloidogenicity of pathogenic lysozyme mutations. *J. Mol. Biol.* 346:773–788.
16. Kumita, J. R., R. J. Johnson, ..., C. M. Dobson. 2006. Impact of the native-state stability of human lysozyme variants on protein secretion by *Pichia pastoris*. *FEBS J.* 273:711–720.

17. Canet, D., A. M. Last, …, C. M. Dobson. 2002. Local cooperativity in the unfolding of an amyloidogenic variant of human lysozyme. *Nat. Struct. Biol.* 9:308–315.

18. Dhulesia, A., N. Cremades, …, C. M. Dobson. 2010. Local cooperativity in an amyloidogenic state of human lysozyme observed at atomic resolution. *J. Am. Chem. Soc.* 132:15580–15588.

19. Hagan, C. L., R. J. Johnson, …, J. R. Kumita. 2010. A non-natural variant of human lysozyme (I59T) mimics the in vitro behaviour of the I56T variant that is responsible for a form of familial amyloidosis. *Protein Eng. Des. Sel.* 23:499–506.

20. Johnson, R. J., J. Christodoulou, …, C. M. Dobson. 2005. Rationalising lysozyme amyloidosis: insights from the structure and solution dynamics of T70N lysozyme. *J. Mol. Biol.* 352:823–836.

21. Dumoulin, M., A. M. Last, …, C. M. Dobson. 2003. A camelid antibody fragment inhibits the formation of amyloid fibrils by human lysozyme. *Nature.* 424:783–788.

22. Chan, P.-H., E. Pardon, …, M. Dumoulin. 2008. Engineering a camelid antibody fragment that binds to the active site of human lysozyme and inhibits its conversion into amyloid fibrils. *Biochemistry.* 47:11041–11054.

23. De Genst, E., P.-H. Chan, …, M. Dumoulin. 2013. A nanobody binding to non-amyloidogenic regions of the protein human lysozyme enhances partial unfolding but inhibits amyloid fibril formation. *J. Phys. Chem. B.* 117:13245–13258.

24. Buell, A. K., A. Dhulesia, …, C. M. Dobson. 2011. Population of nonnative states of lysozyme variants drives amyloid fibril formation. *J. Am. Chem. Soc.* 133:7737–7743.

25. Frare, E., M. F. Mossuto, …, A. Fontana. 2006. Identification of the core structure of lysozyme amyloid fibrils by proteolysis. *J. Mol. Biol.* 361:551–561.

26. Takano, K., K. Ogasahara, …, K. Yutani. 1995. Contribution of hydrophobic residues to the stability of human lysozyme: calorimetric studies and X-ray structural analysis of the five isoleucine to valine mutants. *J. Mol. Biol.* 254:62–76.

27. Takano, K., J. Funahashi, …, K. Yutani. 1997. Contribution of water molecules in the interior of a protein to the conformational stability. *J. Mol. Biol.* 274:132–142.

28. Funahashi, J., K. Takano, …, K. Yutani. 1999. Contribution of amino acid substitutions at two different interior positions to the conformational stability of human lysozyme. *Protein Eng.* 12:841–850.

29. Mombelli, E., M. Afshar, …, R. Lange. 1997. The role of phenylalanine 31 in maintaining the conformational stability of ribonuclease P2 from *Sulfolobus solfataricus* under extreme conditions of temperature and pressure. *Biochemistry.* 36:8733–8742.

30. Greene, R. F., Jr., and C. N. Pace. 1974. Urea and guanidine hydrochloride denaturation of ribonuclease, lysozyme, α-chymotrypsin, and β-lactoglobulin. *J. Biol. Chem.* 249:5388–5393.

31. Mossuto, M. F., A. Dhulesia, …, X. Salvatella. 2010. The non-core regions of human lysozyme amyloid fibrils influence cytotoxicity. *J. Mol. Biol.* 402:783–796.

32. Chiti, F., and C. M. Dobson. 2009. Amyloid formation by globular proteins under native conditions. *Nat. Chem. Biol.* 5:15–22.

33. Chiti, F., and C. M. Dobson. 2006. Protein misfolding, functional amyloid, and human disease. *Annu. Rev. Biochem.* 75:333–366.

34. Kelly, J. W. 1996. Alternative conformations of amyloidogenic proteins govern their behavior. *Curr. Opin. Struct. Biol.* 6:11–17.

35. Dobson, C. M. 1999. Protein misfolding, evolution and disease. *Trends Biochem. Sci.* 24:329–332.

36. Kumita, J. R., S. Poon, …, C. M. Dobson. 2007. The extracellular chaperone clusterin potently inhibits human lysozyme amyloid formation by interacting with prefibrillar species. *J. Mol. Biol.* 369:157–167.

37. Vendruscolo, M., E. Paci, …, C. M. Dobson. 2003. Structures and relative free energies of partially folded states of proteins. *Proc. Natl. Acad. Sci. USA.* 100:14817–14821.

38. Geyer, M., C. Herrmann, …, H. R. Kalbitzer. 1997. Structure of the Ras-binding domain of RalGEF and implications for Ras binding and signalling. *Nat. Struct. Biol.* 4:694–699.

Biophysical Journal, Volume *111*

Supplemental Information

The Significance of the Location of Mutations for the Native-State Dynamics of Human Lysozyme

Minkoo Ahn, Christine L. Hagan, Ana Bernardo-Gancedo, Erwin De Genst, Francisco N. Newby, John Christodoulou, Anne Dhulesia, Mireille Dumoulin, Carol V. Robinson, Christopher M. Dobson, and Janet R. Kumita

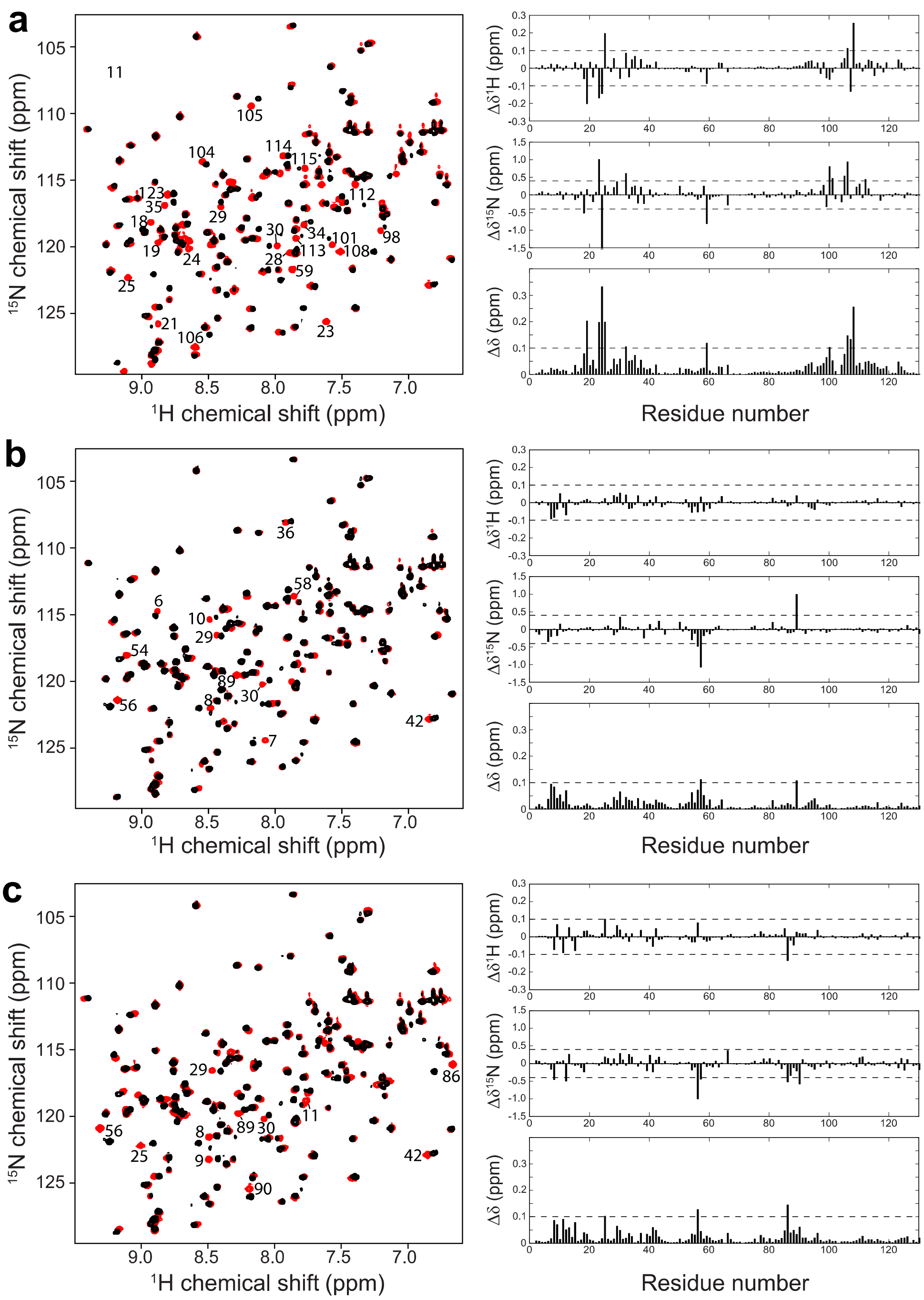

Figure S1. (a), (b), (c) HSQC NMR spectra of I23A (a), I56V (b) and I89V (c) variants (red) overlaid with that of WT lysozyme (black). The spectra were collected at 700 MHz at pH 5.0 and 37 °C. Differences between chemical shift perturbation of each variant and that of WT are shown on the right. $\Delta\delta^{15}N$ (top), $\Delta\delta^{1}H$ (middle) and weighted difference ($\Delta\delta$, bottom) are shown against residue number. Threshold values of ±0.3, ±1.5, ±0.1 for $\Delta\delta^{15}N$, $\Delta\delta^{1}H$ and $\Delta\delta$ are shown in dashed lines. Relative gyromagnetic ratio of ^{15}N and ^{1}H is used as the weighting factor ($\omega = \gamma_{15N}/\ \gamma_{1H}$) for calculating $\Delta\delta = ((\Delta\delta^{1}H)^2 + (\omega\Delta\delta^{15}N)^2)^{0.5}$.

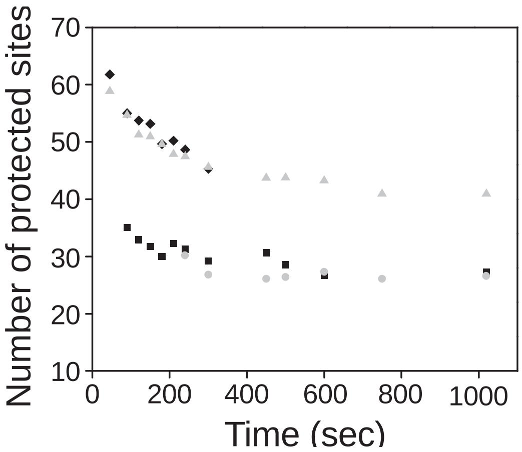

Figure S2. Comparison of the number of protected sites in the higher and lower mass species observed by EX1 HD exchange mass spectrometry for I23A (light grey circles and triangles) and I59T (black diamonds and squares) at pH 8.0, 37°C. Lysozyme variants were deuterated all exchangeable sites and the fully deuterated I23A and I59T had molecular masses of 14912.6 Da and 14943.6 Da respectively. At each time point, the number of exchangeable sites for each mass species was determined (deuterated molecular mass – observed molecular mass). The number of protected sites, i.e. not accessible to the solvent for deuterium-to-hydrogen exchange, for each mass species can be determined (calculated number of exchange sites in variant – observed number of exchanged sites). The calculated number of exchange sites for I23A and I59T are 262 and 263, respectively. Although structural details of the two species cannot be determined under these experimental conditions, it is clear there is close agreement in the number of protected sites for the I23A and I59T variants for both the higher and lower mass species, indicating similar degrees of protection from solvent exchange under EX1 conditions (37°C, pH 8.0).

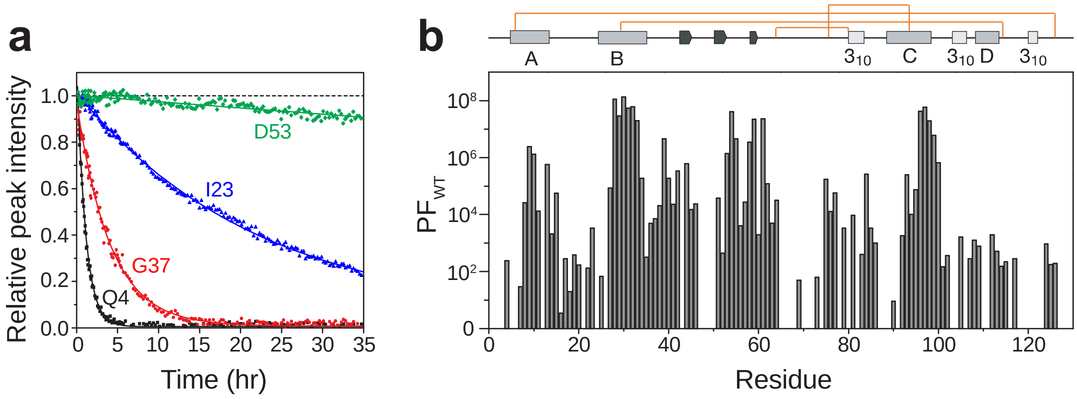

Figure S3. Real-time EX2 HD exchange of the WT protein monitored by NMR. (a) Fitting of peak intensities of four different residues of the WT protein from HSQC spectra to a single exponential decay curve to calculate k_{obs}. (b) Protection factors (PFs) for the amide hydrogen atoms in the WT protein, calculated from the rates of hydrogen exchange monitored by NMR. Residues for which the hydrogen exchange rates are too fast to be measured are left blank.

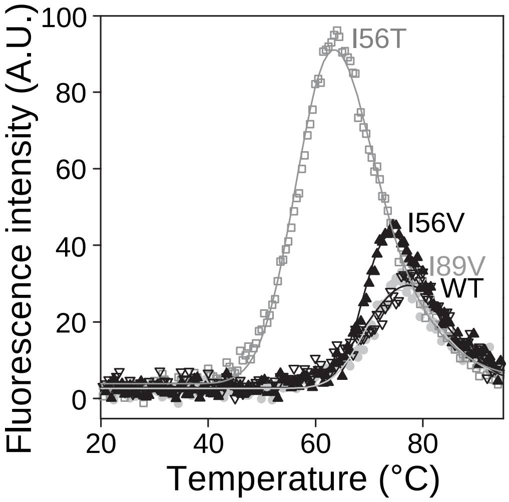

Figure S4. Thermal unfolding of I56V (black, solid triangles) and I89V (light grey circles) monitored by ANS flurosecence emission as in Figure 2(b). All curves were normalised with respect to the maximum fluorescence value of the I56T (dark grey, open squares) variant. Data for I56T and WT (black, open triangles) are shown for comparison.

	$\Delta G^{\circ}(H_2O)_{NU}$ (kJ mol^{-1})	*m* (kJ mol^{-1} M^{-1})	C_m (M)
WT	66.4 ± 3.0 [a]	17.2 ± 0.5 [a]	3.9 ± 0.1 [a]
I59T	39.3 ± 3.3 [a]	13.1 ± 0.7 [a]	3.0 ± 0.1 [a]
I23A	40.6 ± 3.4	13.9 ± 1.1	2.9 ± 0.1

Table S1. Thermodynamic parameters determined from GdnHCl-induced unfolding. [a]Data from (19).
